# TRPV4-dependent induction of a novel mammalian cold-inducible protein SRSF5 as well as CIRP and RBM3

**DOI:** 10.1038/s41598-017-02473-x

**Published:** 2017-05-23

**Authors:** Takanori Fujita, Hiroaki Higashitsuji, Hisako Higashitsuji, Yu Liu, Katsuhiko Itoh, Toshiharu Sakurai, Takahiro Kojima, Shuya Kandori, Hiroyuki Nishiyama, Motoi Fukumoto, Manabu Fukumoto, Koji Shibasaki, Jun Fujita

**Affiliations:** 1 Department of Clinical Molecular Biology, Graduate School of Medicine, Kyoto University, Kyoto, Kyoto, 606-8507 Japan; 2 School of Economics, Nagoya University, Nagoya, Nagoya, 464-8601 Japan; 3 Department of Gastroenterology and Hepatology, Kindai University Faculty of Medicine, Osaka-Sayama, Osaka 589-8511 Japan; 4 Department of Urology, Faculty of Medicine, University of Tsukuba, Tsukuba, Ibaraki 305-8575 Japan; 5 Department of Pathology, Institute of Development, Aging and Cancer, Tohoku University, Sendai, Miyagi 980-8575 Japan; 6 Department of Molecular Pathology, Tokyo Medical University, Shinjuku-ku, Tokyo, 160-8402 Japan; 7 Department of Molecular and Cellular Neurobiology, Gunma University Graduate School of Medicine, Maebashi, Gunma, 371-8511 Japan; 8 Department of Rehabilitation Medicine, Biwako-Chuo Hospital, Otsu, Shiga, 520-0834 Japan

## Abstract

Cold-inducible RNA-binding protein (CIRP) and RNA-binding motif protein 3 (RBM3) are two evolutionarily conserved RNA-binding proteins that are structurally related to hnRNPs and upregulated in response to moderately low temperatures in mammalian cells. Although contributions of splicing efficiency, the gene promoters activated upon mild hypothermia and the transcription factor Sp1 to induction of CIRP have been reported, precise mechanisms by which hypothermia and other stresses induce the expression of mammalian cold-inducible proteins (CIPs) are poorly understood. By screening the serine/arginine-rich splicing factors (SRSFs), we report that the transcript and protein levels of SRSF5 were increased in mammalian cells cultured at 32 °C. Expression of SRSF5 as well as CIRP and RBM3 were also induced by DNA damage, hypoxia, cycloheximide and hypotonicity. Immunohistochemical studies demonstrated that SRSF5 was constitutively expressed in male germ cells and the level was decreased in human testicular germ cell tumors. SRSF5 facilitated production of p19 H-RAS, and increased sensitivity to doxorubicin in human U-2 OS cells. Induction of CIPs was dependent on transient receptor potential vanilloid 4 (TRPV4) channel protein, but seemed independent of its ion channel activity. These findings indicate a previously unappreciated role for the TRP protein in linking environmental stress to splicing.

## Introduction

The transient receptor potential (TRP) channels are, in general, non-selective cation channels that open in response to changes in temperature, ligand binding and other alterations of the channel protein^1, 2^. They play major roles in a variety of sensory modalities such as vision, thermosensation, olfaction, hearing, taste sensation, and mechanosensation, allowing animals to perceive the external environment. Mammalian TRP channels comprise 28 members and are divided into six subfamilies: TRPC, TRPM, TRPV, TRPA, TRPP and TRPML based on their homology of amino acid sequences^3, 4^. Among TRP channels, the temperature-activated kind constitute a subgroup formed by TRPV1–4, TRPM2, 4, 5, 8, TRPC5, and TRPA1^5, 6^. All of these thermo-TRPs can be activated within specific temperature ranges and transduce inputs into chemical and electrical signals. TRPV3 and TRPV4 have been proposed to be molecular sensors that are involved in the detection of non-noxious warmth. In heterologous expression systems, these channels show a steep increase in activation in response to increases in temperature between 25 and 35 °C^6^. Interestingly, these temperatures include those known to induce mammalian cold-inducible proteins (CIPs), CIRP (Cold-inducible RNA-binding protein, also called CIRBP or A18 hnRNP) and RBM3 (RNA-binding motif protein 3)^7, 8^.

CIRP and RBM3 are the first proteins found to be induced by mild hypothermia in mammalian cells^9^. These proteins are highly similar to each other and belong to the glycine rich RNA-binding protein family class IVa which is characterized by an RNA recognition motif (RRM, also called CS-RBD) at the N-terminus and a glycine-rich domain at the C-terminus^10^. CIRP and RBM3 are constitutively expressed in the testis the temperature of which is physiologically lower than the body cavity temperature^11–13^. In addition to mild hypothermia, CIRP is inducible by other stimuli such as UV and hypoxia, and involved in spermatogenesis, UV-resistance, anti-apoptosis, cell cycle progression and anti-senescence^9^. Furthermore, recent reports have demonstrated that CIRP is a regulator of circadian oscillator genes, including the *CLOCK* gene^14^, and that when present outside the cells CIRP functions as a damage-associated molecular pattern (DAMP) molecule promoting inflammatory responses and tumorigenesis^15, 16^. Like CIRP, RBM3 is inducible by mild hypothermia and hypoxia. In general, RBM3 enhances global protein translation, and is believed to be a pleiotropic regulator of miRNA and mRNAs^9^. Although possible involvement of the transcription factor Sp1, the promoters in the *CIRP* gene activated upon mild hypothermia, and importance of splicing efficiency in induction of CIRP have been reported^17–19^, precise mechanisms by which hypothermia and other stresses induce the expression of CIPs are poorly understood. Especially not clarified are the temperature sensors and the signaling pathways leading to the increased expression.

Serine/arginine (SR)-rich proteins (also called SR splicing factors, SRSFs) and heterogeneous nuclear ribonucleoproteins (hnRNPs) are two important families of splicing factors that activate or repress splice site selection^20, 21^. SR proteins are characterized by one or two N-terminal RRMs, followed by a downstream domain rich in arginine and serine residues, the RS domain^22^. The mammalian SR protein family consists of 12 members (SRSF1-12), and that of hnRNP at least 24^20–22^. The commonalities between these factors consist of the presence of the RRM and their modular domain structure. An ancestral arginine-rich C-terminus seems to have evolved into the canonical RS domain of SR proteins, and hnRNPs contain, in many cases, RGG boxes, suggesting that SR proteins and hnRNPs have a common ancestor^23^. As both CIRP and RBM3 are structurally similar to hnRNPs with RRM and RGG domains, and associate with the spliceosome^9^, we tried to determine in this study whether there are any CIPs belonging to SR proteins. We found that SRSF5 (also called SRp40 or SFRS5) protein is inducible by mild hypothermia and other stresses like CIRP and RBM3. Furthermore, we provide evidence that TRPV4 is necessary for the induction. These findings indicate a previously unappreciated role for the TRP channel protein in linking environmental stress to splicing.

## Results

### SRSF5 is a novel mammalian CIP

**Figure 1:**
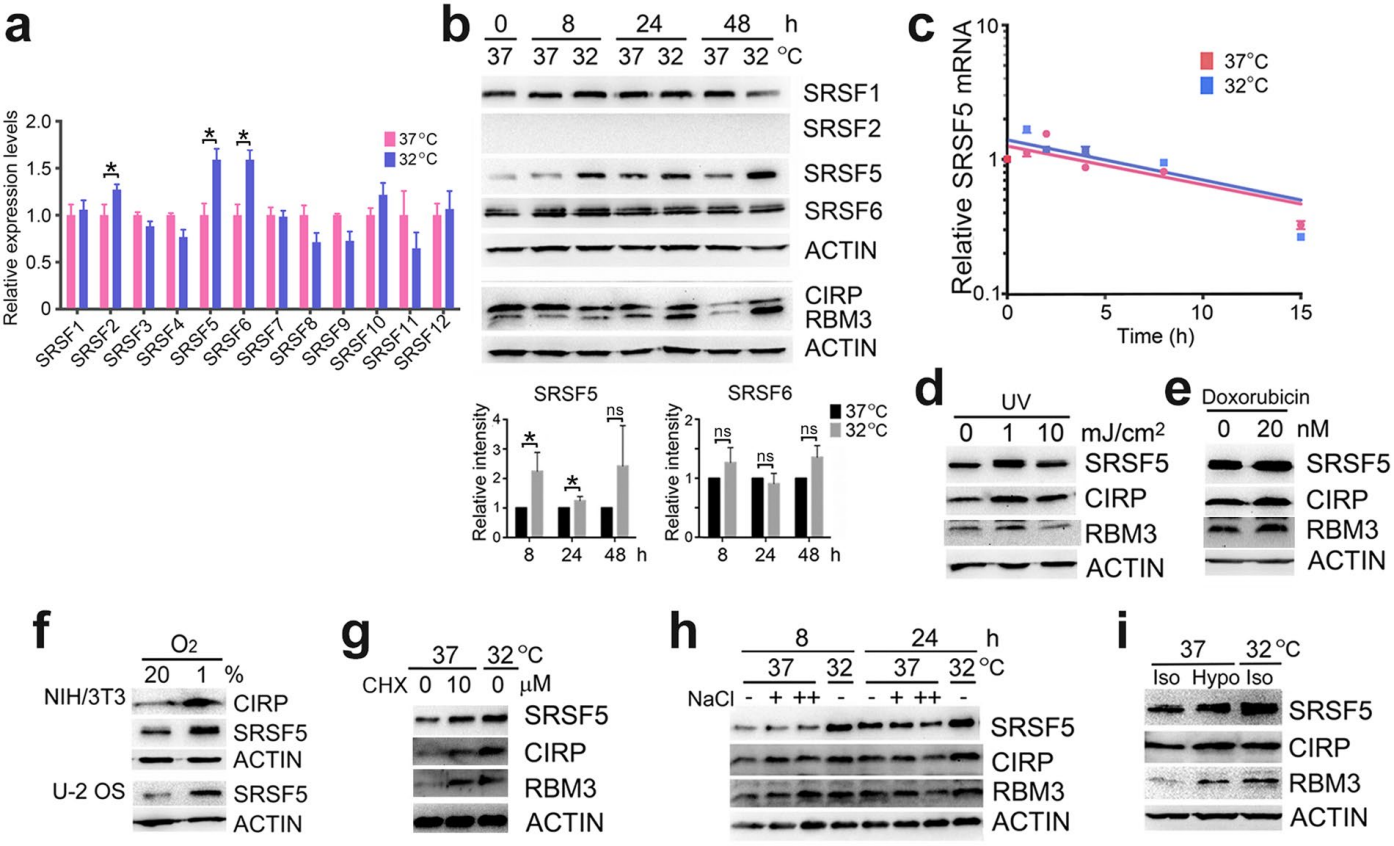
Induction of SRSF5 protein by various stresses. (**a**) Comparison of transcript levels of SRSF family members (1 to 12) in U-2 OS cells cultured at 37 °C or 32 °C for 6 h. mRNA abundance at 32 °C relative to that at 37 °C after normalization to 18S rRNA was determined for each SRSF member by quantitative RT-PCR (data indicate mean ± SEM; n = 3 per group). Statistical significance was determined by Student’s *t*-test. *, *P* < 0.05. (**b**) Protein levels in U-2 OS cells cultured at 37 °C or 32 °C for indicated times were analyzed by western blot (upper panels, representative results). Band intensities relative to those at 37 °C were determined after normalization to ACTIN (lower graphs, data indicate mean ± SEM; n = 3). The samples derived from the same experiment and gels/blots were processed in parallel. Statistical significance was determined by Student’s *t*-test. *, *P* < 0.05. ns, *P* > 0.05. (**c**) U-2 OS cells were cultured at 37 °C or 32 °C in the presence of 5 μg/ml actinomycin D for indicated times. SRSF5 mRNA levels relative to those at time 0 were determined by quantitative RT-PCR after normalization to 18S rRNA (data indicate mean ± SEM; n = 3). (**d** and **e**) U-2 OS cells were cultured at 37 °C for 8 h after exposure to indicated doses of UV (**d**) or in the presence or absence of 20 nM doxorubicin (**e**). Cell lysates were analyzed by western blot (representatives of 4 or 3 independent experiments). (**f**) NIH/3T3 and U-2 OS cells were cultured at 37 °C under normoxia (20%) or hypoxia (1% O_2_) for 8 h, and analyzed by western blot (representative of 4 independent experiments). (**g**) U-2 OS cells were cultured at 37 °C for 12 h in the presence of indicated doses of cycloheximide (CHX), and analyzed by western blot (representative of 5 independent experiments). (**h**) U-2 OS cells were incubated with regular media (−) or media containing additional 60 (+) or 160 (++) mM NaCl at 37 °C for 8 or 24 h, and analyzed by western blot (representative of 4 independent experiments). (**i**) U-2 OS cells were incubated with regular media (Iso) or hypotonic media containing 10% (volume/volume) of H_2_O (Hypo) at 37 °C for 8 h, and analyzed by western blot (representative of 3 independent experiments). Full-length blots are presented in Supplementary Fig. 6.

Six hours after transfer from 37 °C to 32 °C culture temperature, transcript levels of 3 out of 12 members of SRSF family, namely, SRSF2, SRSF5 and SRSF6, were significantly increased in human U-2 OS cells (Fig. 1a). At the protein level, SRSF5, but not SRSF6, was increased, and no SRSF2 band could be detected under the present conditions (Fig. 1b). SRSF5 protein was induced at 8 h after transfer, and then the level decreased slightly. Hypothermia also induced SRSF5 protein in mouse embryonic fibroblasts derived from wild-type and *CIRP*-knockout (KO) mice, NIH/3T3 cells, human HEK293 cells and NC65 cells (Supplementary Fig. S1).

To compare stability of SRSF5 mRNA at 37 °C and 32 °C, we treated U-2 OS cells with actinomycin D to prevent synthesis of new transcripts. SRSF5 mRNA half-life remained nearly the same at both temperatures (Fig. 1c), suggesting that the induction is transcriptional.

CIRP orthologue was independently identified as one of the UV-inducible proteins in hamster cells^24^. SRSF5 as well as CIRP were induced by UV in U-2 OS cells (Fig. 1d and Supplementary Fig. S1). When DNA damage was induced by doxorubicin, induction of CIPs was also observed (Fig. 1e and Supplementary Fig. S1).

Like CIRP and RBM3^25^, hypoxia induced SRSF5 in NIH/3T3 cells and U-2 OS cells (Fig. 1f and Supplementary Fig. S1).

Previously, we reported induction of RBM3 by cycloheximide^8^. Cycloheximide induced all three CIPs (Fig. 1g and Supplementary Fig. S1).

In fish, CIRP homologues are induced upon environmental hyperosmotic stress^26^. In U-2 OS cells, increasing osmolality by 160 mOsm did not induce the expression of CIPs (Fig. 1h and Supplementary Fig. S1). By contrast, exposure to hypotonic media containing 10% (volume/volume) of H_2_O for 8 h increased the CIP levels (Fig. 1i and Supplementary Fig. S1). Exposure to hypotonic media for 2 h was sufficient to induce SRSF5 at 8 h, but not for CIRP and RBM3 (Supplementary Fig. S1).

### Subcellular localization and testicular expression of SRSF5 protein

**Figure 2:**
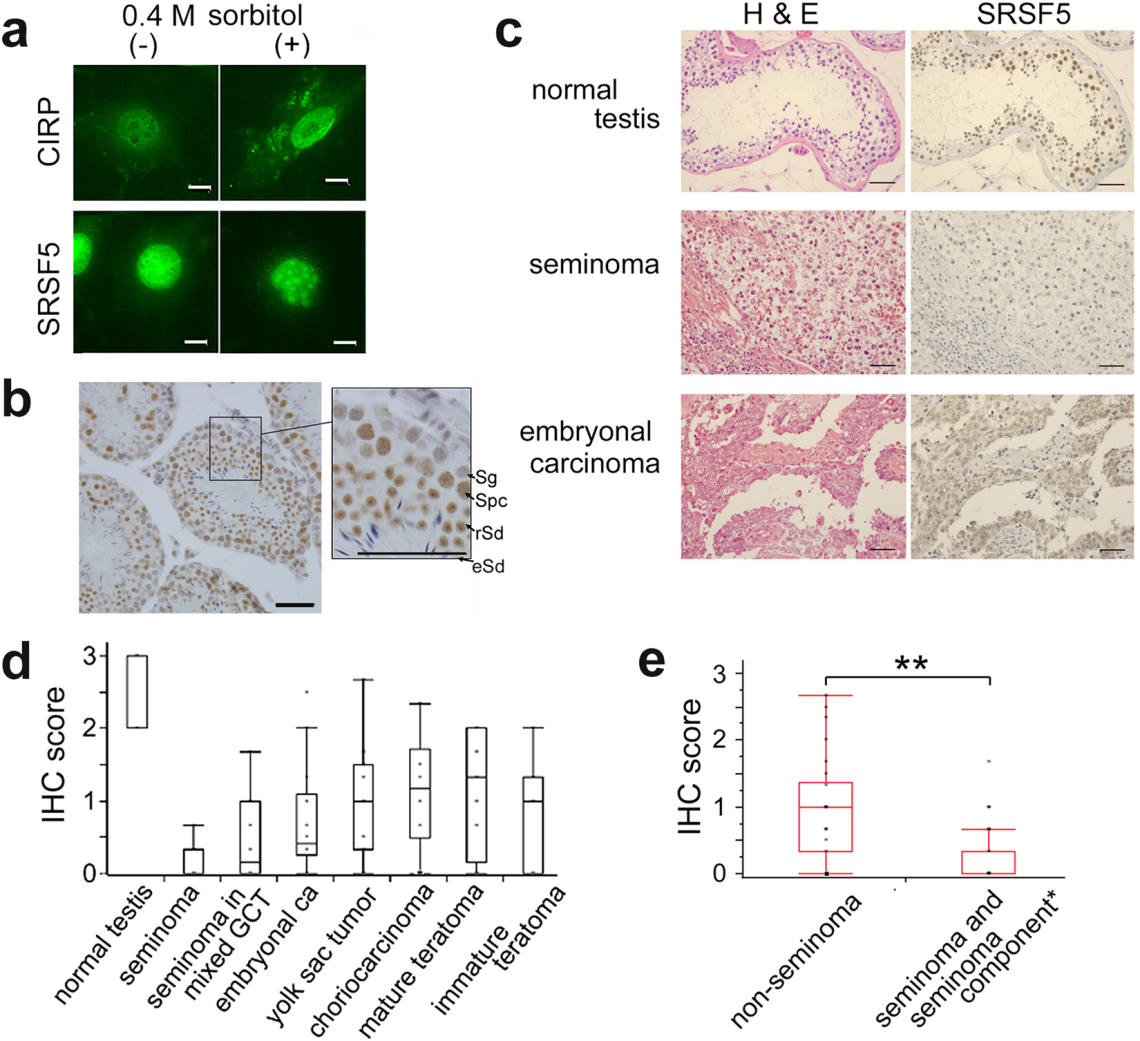
Subcellular localization and testicular expression of SRSF5 protein. (**a**) Localization of SRSF5 and CIRP proteins detected by immunofluorescence microscopy in NIH/3T3 cells cultured in PBS with (+) or without (−) 0.4 M sorbitol at 37 °C for 2 h. Scale bars, 50 μm. (**b**) Immunohistochemical (IHC) detection of SRSF5 protein in the testis of 6-wk-old C57BL/6 J mouse. Square region was enlarged in the right panel. Sg, spermatogonia; Spc, spermatocytes; rSd, round spermatids; eSd, elongated spermatids. Scale bars, 50 μm. (**c**) Hematoxylin and eosin (H&E) staining and IHC staining for SRSF5 of human normal testis and testicular germ cell tumors. Representative results are shown. Scale bars, 50 μm. (**d**) IHC scoring of SRSF5 protein levels in human normal testes and germ cell tumors (data indicate mean ± SEM. See Methods for the numbers of samples). (**e**) IHC comparison of SRSF5 protein levels between non-seminomas (n = 19) and seminoma components of mixed tumors and pure seminomas (n = 30) (data indicate mean ± SEM). Statistical significance was determined by Student’s *t*-test. **, *P* < 0.01.

Methylation of arginine residues in the RGG domain of CIRP due to cytoplasmic or ER stress causes CIRP accumulation in cytoplasmic stress granules^27^. Although exposure of NIH/3T3 cells to osmotic shock (PBS + 400 mM sorbitol) induced a detectable accumulation of CIRP in cytoplasmic stress granules, SRSF5 remained in the nucleus and accumulated in nuclear speckles (Fig. 2a).

During hemorrhagic shock and sepsis, CIRP is released from the heart and liver and acts as a DAMP^15^. In macrophages under hypoxic stress *in vitro*, CIRP translocates from the nucleus to the cytosol and is released. In accord with this, we detected CIRP in culture supernatants of THP-1 cells, a human macrophage cell line (Supplementary Fig. S2). However, no SRSF5 was detected even under hypoxic conditions.

Since CIRP and RBM3 are highly expressed in testicular germ cells and Sertoli cells, respectively^11, 12^, expression of SRSF5 protein in the mouse testis was examined by immunohistochemistry (Fig. 2b). Strong signals were present in the nuclei of pachytene spermatocytes and round spermatids. No significant signals were detected in spermatogonia or elongated spermatids. Faint signals were detected in Sertoli cells.

Immunohistochemistry of normal human testis showed that SRSF5 was expressed dominantly in spermatocytes and spermatids (Fig. 2c). In testicular germ cell tumors, expression of SRSF5 protein was higher in non-seminomas than pure seminomas and the seminoma components of mixed germ cell tumors (*P* < 0.0001), although the levels were lower than that of normal germ cells (Fig. 2c–e and Supplementary Fig. S2). Expression of SRSF5 was not associated with metastasis in seminomas or embryonal carcinomas (Supplementary Fig. S2).

### Effects of SRSF5 on cell proliferation

**Figure 3:**
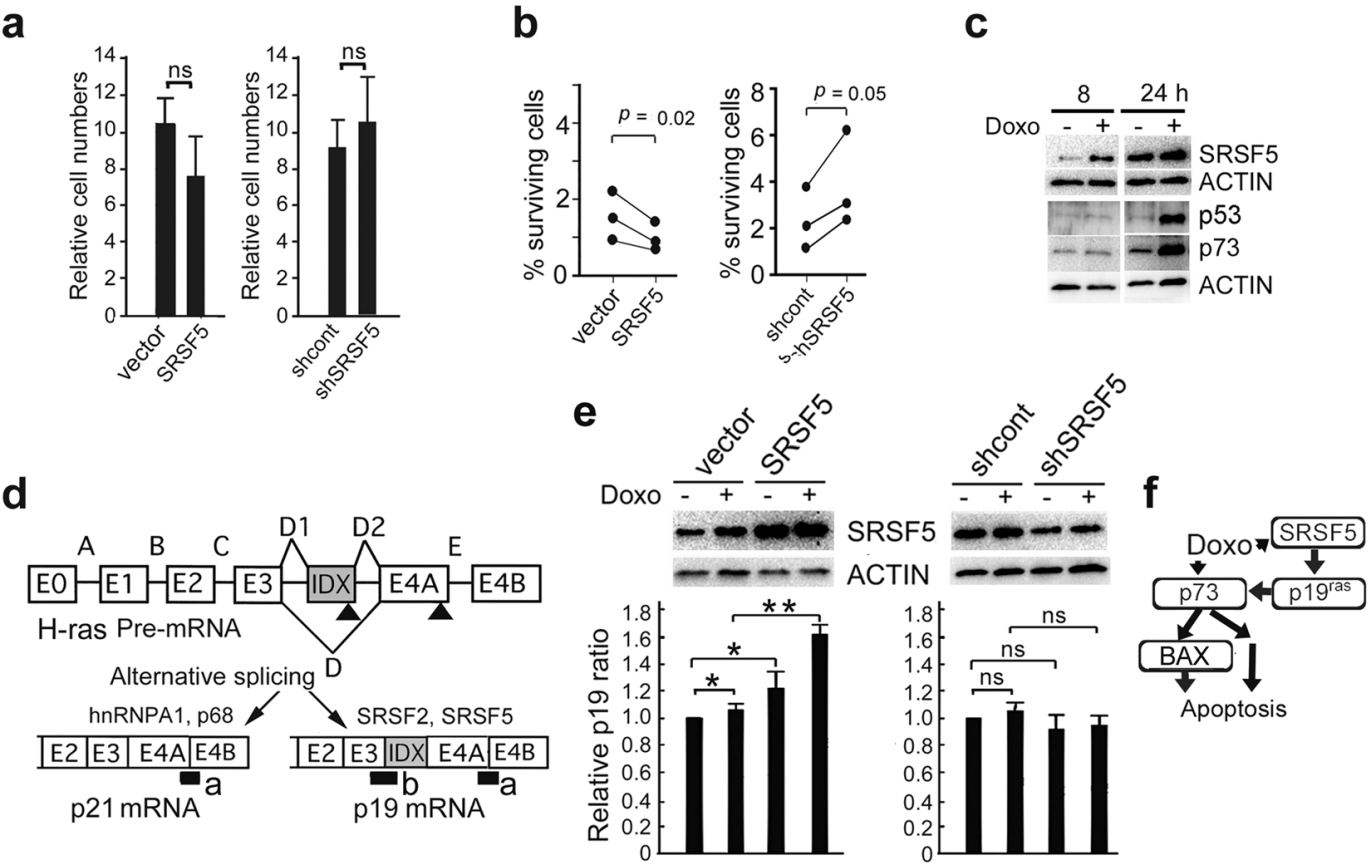
Effects of SRSF5 on cell proliferation. (**a**) Stable transfectants of U-2 OS cells overexpressing SRSF5, vector alone, shRNA control and shRNA against SRSF5 (shSRSF5) were cultured at 37 °C for 4 days. Cell numbers were determined and expressed as relative to those at day 0 (mean ± SEM; n = 3). ns, *P* > 0.05. (**b**) Transfectants were cultured at 37 °C in the presence of 100 nM doxorubicin (Doxo) for 4 days. Numbers of surviving cells were determined and compared with those of untreated cells (data indicate mean ± SEM; n = 3). (**c**) U-2 OS cells were cultured at 37 °C in the presence (+) or absence (−) of 100 nM Doxo for 8 or 24 h, and analyzed by western blot (representative of 4 independent experiments). (**d**) Scheme of the Taqman assays used to determine endogenous p19 and total (p19 and p21) *H-RAS* mRNA expression. cDNA regions amplified by E4A-E4B and E3-IDX primer pairs are indicated by a and b, respectively. E0 to E4B, exons of human *H-RAS* gene. Arrow heads, stop codons. (**e**) Transfectants were cultured as in (**c**) for 10 h, and analyzed by western blot and the Taqman assays (upper panels, representative results). Relative p19 ratio, the p19/total *H-RAS* mRNA ratio obtained for each sample after normalization to 18S rRNA, and expressed as relative to that of control cells cultured without Doxo (mean ± SEM; n = 3). *, *P* < 0.05. **, *P* < 0.01. ns, *P* > 0.05. (**f**) A model how SRSF5 enhances apoptosis induced by Doxo. Statistical significance was determined by Student’s *t*-test. Full-length blots are presented in Supplementary Fig. 6.

To evaluate SRSF5 functions, we generated stable clones of SRSF5-overexpressing and SRSF5-knockdown U-2 OS cells together with respective controls (Supplementary Fig. S3). Modulating SRSF5 protein levels did not significantly affect cell proliferation (Fig. 3a). When U-2 OS cells were exposed to doxorubicin, however, U-2 OS cells overexpressing SRSF5 survived less than control cells (Fig. 3b, left). Consistently, more SRSF5-knockdown cells survived than controls cells (Fig. 3b, right).

Similar to p53, p73 accumulates in response to DNA damaging agents and plays a critical role in induction of apoptotic cell death^28^. p53 and p73 were induced by doxorubicin following SRSF5 in U-2 OS cells (Fig. 3c and Supplementary Fig. S3). *H-RAS* pre-mRNA can be alternatively spliced in the IDX and 4 A terminal exons, yielding the p19 and p21 proteins, respectively (Fig. 3d)^29^. SRSF2 and SRSF5 are know to promote production of p19^30^. p19 interacts with p73 and activates its transcriptional activity, leading to increased pro-apoptotic BAX expression and cytochrome c release^31^. When U-2 OS cells were treated with doxorubicin, p19 mRNA level was higher in cells overexpressing SRSF5 (Fig. 3e). Consistently, BAX levels was higher in them (Supplementary Fig. S3). Thus, SRSF5 was induced by doxorubicin and enhanced production of p19 protein, which, in combination with p73, could sensitize U-2 OS cells to cytotoxic effects of doxorubicin (Fig. 3f).

### Regulatory mechanisms of SRSF5 induction

**Figure 4:**
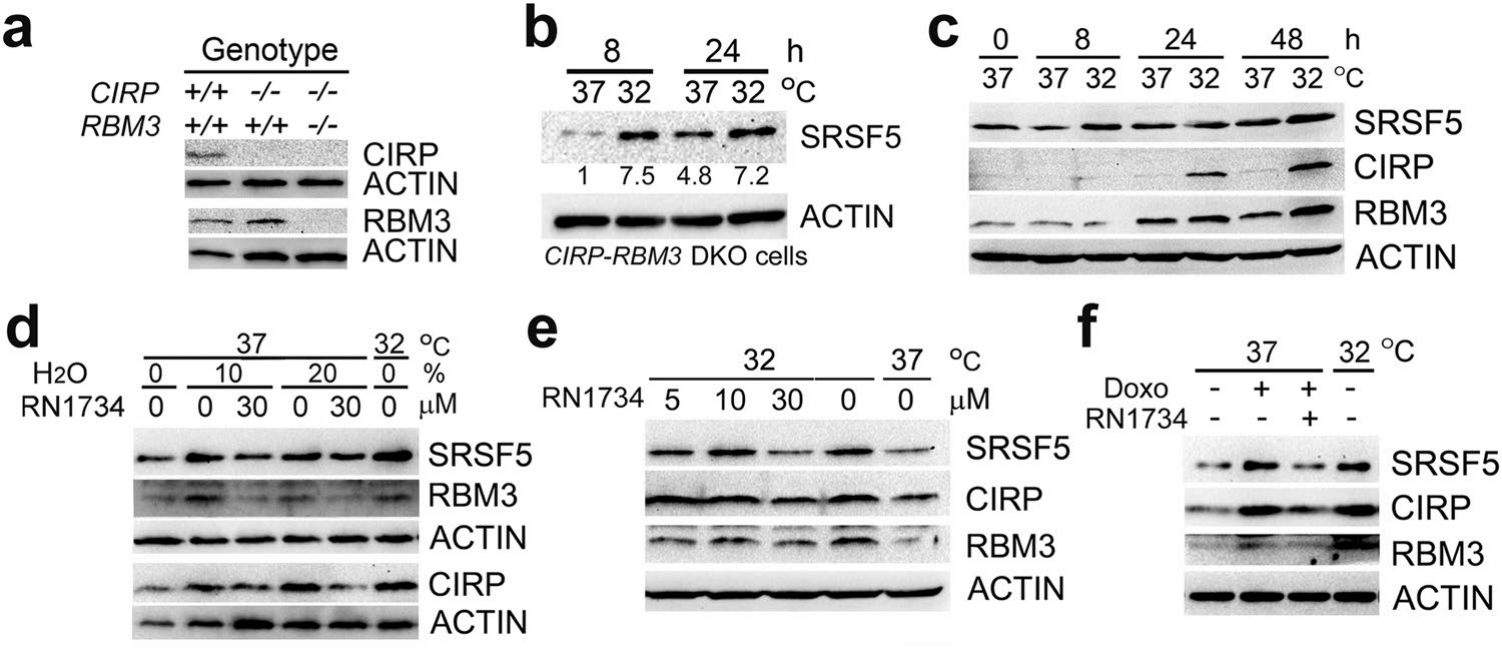
Regulatory mechanisms of SRSF5 induction. (**a** and **b**) Fibroblasts from wild-type (+/+) or knockout (−/−) mice were analyzed for expression of CIRP and RBM3 by western blot (representative of 2 independent experiments) (**a**). *CIRP-RBM3* double knockout (DKO) fibroblasts were cultured at 37 °C or 32 °C for 8 or 24 h, and analyzed by western blot. Relative band intensities after normalization to ACTIN expression are shown below the panel (representative of 2 independent experiments) (**b**). (**c**) B22 cells were cultured at 37 °C or 32 °C for indicated times, and analyzed by western blot (representative of 3 independent experiments). (**d**) U-2 OS cells were incubated with isotonic regular media (0%) or hypotonic media containing 10 or 20% (volume/volume) of H_2_O at 37 °C or 32 °C in the presence of 0 or 30 μM RN1734 for 8 h, and analyzed by western blot (representative of 3 independent experiments). (**e**) U-2 OS cells were cultured at 37 °C or 32 °C in the presence of indicated concentrations of RN1734 for 24 h, and analyzed by western blot (representative of 3 independent experiments). (**f**) U-2 OS cells were cultured at 37 °C or 32 °C in the absence (−) or presence (+) of 30 μM RN1734 together with 20 nM doxorubicin (Doxo) for 8 h, and analyzed by western blot (representative of 4 independent experiments). Full-length blots are presented in Supplementary Fig. 6.

CIRP and RBM3 are RNA-binding proteins that can modulate RNAs at the post-transcriptional level^9^. To explore the possibility that enhanced expression of SRSF5 mRNA and protein was due to activities of CIRP and/or RBM3, we used fibroblasts derived from *CIRP-RBM3* double knockout (KO) mice (Fig. 4a). As shown in Fig. 4b, incubation at 32 °C induced expression of SRSF5 in double KO cells, demonstrating that the presence of CIRP or RBM3 is not necessary for the induction.

Like hypothermia, most stresses that induce the mammalian CIPs also suppress cell proliferation. Therefore, we isolated a subclone of mouse BALB/3T3 cells (B22 cells) that proliferates well at 32 °C (Supplementary Fig. S4), and subjected this clone to hypothermia. SRSF5, CIRP and RBM3 were induced in B22 cells after transfer to 32 °C (Fig. 4c and Supplementary Fig. S4), indicating that suppression of proliferation did not cause induction of these proteins.

### TRPV4-dependent induction of CIPs

Among thermo-TRPs responding to warm temperatures, TRPV4, but not TRPV3 channel is activated by extracellular hypotonicity^32, 33^. We, therefore, suspected an involvement of TRPV4 in the induction of CIPs by hypotonic stress. A TRPV4 antagonist, RN1734, inhibited induction of CIPs by hypotonic stress (Fig. 4d and Supplementary Fig. S4). RN1734 suppressed induction of all CIPs by hypothermia, although it stimulated expression of SRSF5 at 37 °C (Fig. 4e and Supplementary Fig. S4). RN1734 also suppressed the induction by doxorubicin, but the effects were absent or weak on induction by UV, hypoxia and cycloheximide (Fig. 4f and Supplementary Fig. S4).

**Figure 5:**
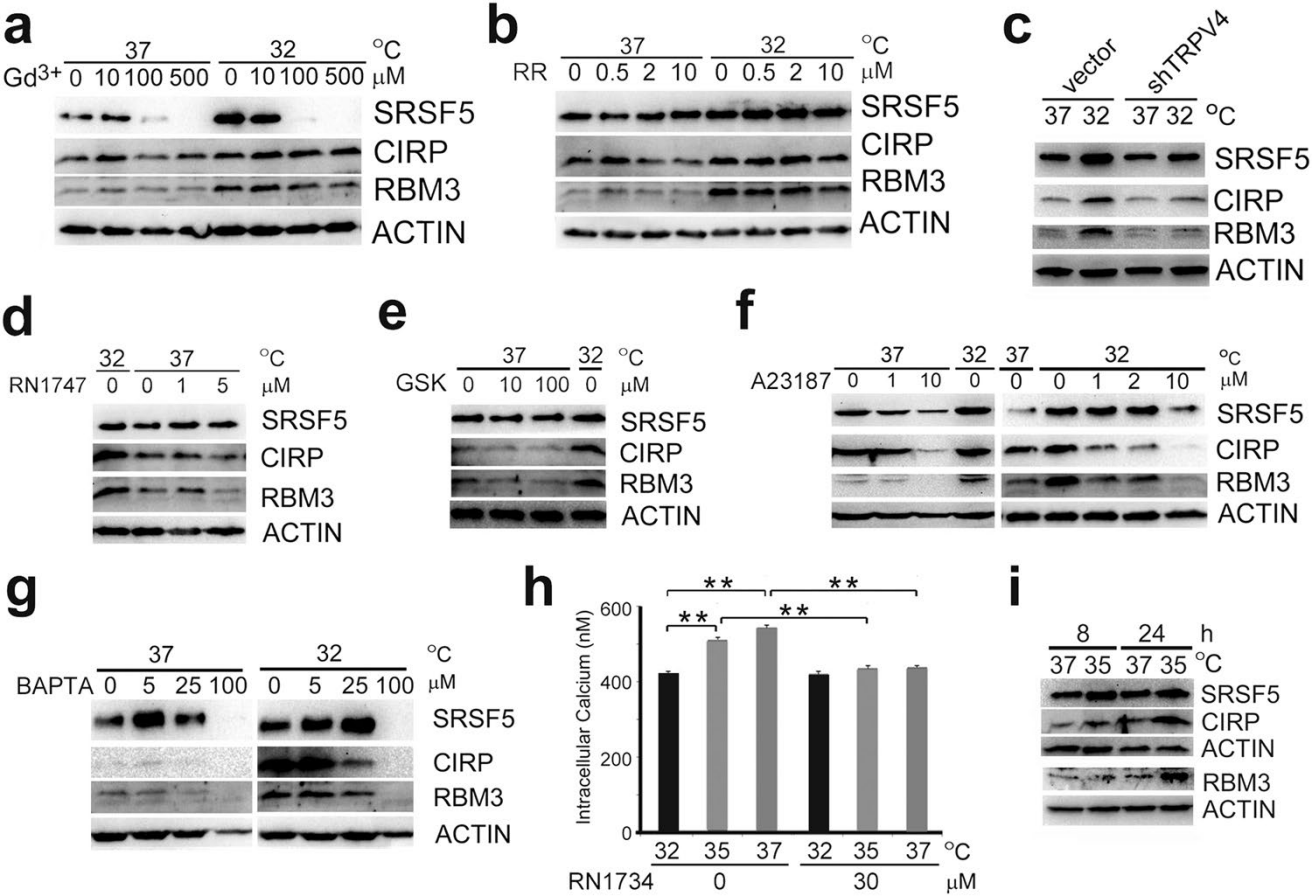
TRPV4 ion channel activity and induction of CIPs. (**a** and **b**) U-2 OS cells were cultured at 37 °C or 32 °C for 24 h in the presence of indicated doses of gadolinium chloride (Gd^3+^) (**a**) or ruthenium red (RR) (**b**), and analyzed by western blot (representatives of 3 independent experiments each). (**c**) U-2 OS transfectants transiently expressing vector alone or shRNA against TRPV4 (shTRPV4) were cultured at 37 °C or 32 °C for 16 h, and analyzed by western blot (representative of 5 independent experiments). (**d,e,f** and **g**) U-2 OS cells were cultured at 37 °C or 32 °C for 24 h in the presence of indicated doses of RN1747 (**d**), GSK1016790A (**e**), A23187 (**f**), or BAPTA-AM (**g**), and analyzed by western blot (representatives of 3 independent experiments each). (**h**) Quantification of intracellular Ca^2+^ concentrations by Fura-2 in U-2 OS cells. Cells were cultured at 37 °C, 35 °C or 32 °C for 24 h in the presence or absence of 30 μM RN1734 (data indicate mean ± SEM; n = 6). Statistical significance was determined by Student’s *t*-test. **, *P* < 0.01. (**i**) U-2 OS cells were cultured at 37 °C or 35 °C for indicated times, and analyzed by western blot (representative of 4 independent experiments). Full-length blots are presented in Supplementary Fig. 6.

In general, TRPV channels are inhibited by Gd^3+^ ^34^. Above 100 μM, gadolinium chloride suppressed induction of SRSF5, but not CIRP or RBM3 (Fig. 5a and Supplementary Fig. S5). Ruthenium red (RR), a broad inhibitor of nonselective cation channels including all TRP channels^34^, showed almost no effect up to 10 μM on induction of CIPs (Fig. 5b and Supplementary Fig. S5). Although RN 1734 displays selectivity for TRPV4 over other TRP channels, it may have additional effects on other molecule(s) essential for the induction of CIPs. To exclude this possibility, we knocked down endogenous TRPV4 mRNA by expressing shRNA. In the TRPV4-knockdown U-2 OS cells, but not TRPA1-knockdown U-2 OS cells, induction by hypothermia of all CIPs were suppressed (Fig. 5c and Supplementary Fig. S5). Similar effects of TRPV4-knockdown on induction of CIPs were observed in HEK293 cells (Supplementary Fig. S5). Since hypothermia did not affect the mRNA level of TRPV4 in U-2 OS cells (Supplementary Fig. S5) whereas the mRNA levels for CIPs increased about 2 folds, we assumed that activation of the TRPV4 channel mainly mediates induction of CIPs.

### TRPV4 ion channel activity and induction of CIPs

A TRPV4 agonist, RN1747, mimicked the effects of hypothermia on CIPs in U-2 OS cells (Fig. 5d and Supplementary Fig. S5). Unexpectedly, a more potent and specific TRPV4 agonist, GSK1016790A, did not induce expression of CIPs (Fig. 5e and Supplementary Fig. S5). GSK1016790A did not induce CIPs in HEK293cells, either (Supplementary Fig. S5).

TRPV4 mediates heat-evoked calcium influx^35^. The calcium ionophore A23187, however, did not induce, but rather inhibited expression of CIPs both at 37 °C and 32 °C (Fig. 5f and Supplementary Fig. S5). BAPTA-AM, a cell-permeant chelator which buffers intracellular calcium, increased the expression of CIPs at 5 μM both at 37 °C and 32 °C (Fig. 5g and Supplementary Fig. S5). These results suggest that an increase in intracellular Ca^2+^ ([Ca^2+^]i) caused by activation of TRPV4 ion channel could suppress induction of CIPs.

When HEK293 cells stably expressing exogenous TRPV4 were cultured at 37 °C and 32 °C, [Ca^2+^]i was higher in the former than the latter, which was not observed in the presence of a TRPV4 antagonist HC067047 (Supplementary Fig. S5). Similarly, when U-2 OS cells expressing endogenous TRPV4 were cultured at 37 °C, 35 °C and 32 °C, [Ca^2+^]i was highest at 37 °C and lowest at 32 °C, and the differences were not observed in the presence of RN1734 (Fig. 5h). These results indicate that the observed differences in [Ca^2+^]i between them resulted predominantly from the activation of calcium influx through TRPV4 at 35 °C and 37 °C.

Although TRPV4 channel is supposed to be open at both 37 °C and 35 °C^35^, temperature down-shift from 37 °C to 35 °C efficiently induced expression of CIPs (Fig. 5i and Supplementary Fig. S5).

## Discussion

SRSF5 is a splicing regulator, reported as an insulin-induced protein in regenerating liver, a TGF-β1-induced splicing factor that enhances EDA exon inclusion in fibronectin mature mRNA, and a major regulator of human immunodeficiency virus type 1 mRNA splicing^36–38^. Here, we found that SRSF5 mRNA is induced by lowering culture temperature by 2 °C. During erythroid cell differentiation, SRSF5 protein decreases drastically despite a concomitant upregulation of SRSF5 mRNA level^39^. During hypothermia, SRSF5 protein level was increased as well. Although CIRP and RBM3 are RNA-binding proteins and able to affect mRNA metabolism, induction of SRSF5 by hypothermia is independent of CIRP and RBM3 since it was observed in cells deficient in both CIRP and RBM3. Thus, SRSF5 is a new member of the mammalian CIPs.

Both CIRP and RBM3 are considered to be oncoproteins^40^, but their roles in clinical cancer seem to be opposite. RBM3 and CIRP expression correlates with good and poor prognosis, respectively^9^. SRSF5 expression is increased, associated with lymph node metastasis, and involved in the production of the anti-apoptotic form of Mcl-1 in breast cancers^41^. SRSF5 also stimulates proliferation of lung cancer cells^42^. In HCC, however, SRSF5 is downregulated^43^. In the present study, the SRSF5 protein level was low in the testicular germ cell tumors compared with normal germ cells, and no correlation was observed with metastasis. In U-2 OS cells SRSF5 increased sensitivity to doxorubicin toxicity, at least partly, by facilitating expression of p19 H-RAS protein and promoting apoptosis.

Little is known about regulation of SRSF5 expression. We found that it is inducible by UV, doxorubicin, hypoxia and cycloheximide like CIRP and RBM3. The induction is unrelated to suppression of cell proliferation as hypothermia induced SRSF5 in B22 cells proliferating at 32 °C as efficiently as 37 °C.

Hyperosmolarity enhances expression of the CIRP homologue in salmon^26^. In contrast, in Arabidopsis, expression of the CIRP homologue AtGRP7 is suppressed by hyperosmolarity^44^. Here, hypertonic stress did not induce CIPs in mammalian cells, but hypotonic stress did. The signaling mechanisms leading to induction may not be exactly the same among CIPs because exposure to hypotonic media for 2 h was sufficient to induce SRSF5, but not for CIRP and RBM3.

Induction of CIPs by hypothermia was suppressed by a selective TRPV4 antagonist RN1734 and the shRNA against TRPV4, demonstrating that TRPV4 is necessary for the induction. TRPV4 is widely distributed and was proposed to sense temperatures in the hypothalamus, skin and primary sensory neurons^45, 46^. A more recent study, however, has shown that even *TRPV3*/*TRPV4* double KO mice do not have a deficit in warmth sensitivity, arguing against a general role for TRPV3/TRPV4 in thermosensation^47^. The present results suggest that TRPV4 might play a role in stress response to warm temperatures by mediating or initiating induction of mammalian CIPs.

Although induction of CIPs by hypothermia was dependent on TRPV4, effects of the TRPV4 antagonists and agonists were not consistent with their effects on ion channel activity^34^. The antagonist RN1734 suppressed the induction of CIPs at 32 °C, but induced SRSF5 expression at 37 °C. Gd^3+^ suppressed the induction of SRSF5, but no effect on CIRP and RBM3 was observed. CIPs were induced by hypothermia in the presence of ruthenium red, a non-competitive pan inhibitor of all TRPs. TRPV4 agonist RN1747 induced expression of CIPs. A more specific and potent agonist GSK1016790A, however, showed no inducing activity. Binding of agonists or antagonists as well as hypothermia is expected to cause conformational changes and modifications of TRPV4 protein complexes, and some of them could not affect expression of CIPs despite their effects on ion channel activity.

TRPV4 functions as a nonselective cation channel with moderate calcium selectivity^2, 48^. Calcium ionophores generally facilitate the transport of Ca^2+^ across the plasma membrane, and A23187 mimics the effects of exposure to hypotonic media on extracellular ATP efflux^49^. However, A23187 did not induce, but suppressed expression of CIPs, and a chelator BAPTA-AM induced it, suggesting that increased [Ca^2+^]i does not favor induction of CIPs. By raising ambient temperatures from room temperatures to target temperatures and measuring the ion channel activity, we and others have demonstrated that TRPV4 is activated by warm temperatures (>34 °C)^35, 50, 51^, although one study showed that TRPV4 is activated above 27 °C^52^. Thus, the TRPV4 channel is probably open in cells at 37 °C and 35 °C, and closed at 32 °C. Consistent with this, we observed higher [Ca^2+^]i in TRPV4-expressing cells at 37 °C and 35 °C than 32 °C, and the differences disappeared in the presence of TRPV4 inhibitors. Importantly, we also observed induction of CIPs in cells transferred from 37 °C to 35 °C. Taken together, these results suggest that TRPV4 protein, but not its ion channel activity, is necessary for induction of CIPs at 32 °C.

Therapeutic hypothermia efficiently reduces injury in acute ischemia, and delays the progression of chronic neurodegenerative diseases^53, 54^. *In vitro*, CIRP and RBM3 function against apoptosis in neurons^55, 56^. Hypothermia-induced CIRP expression also protects hepatocytes in fulminant hepatitis by reducing ROS production^57^. On the other hand, anti-CIRP antibody treatment, which neutralizes secreted CIRP, decreases the inflammatory response and protects from hemorrhagic shock, sepsis and the ischemic-reperfusion injury of the liver^15, 58^. By targeting TRPV4 and the signaling pathways leading to induction of CIPs, it might be possible to modulate the expression levels of CIPs and provide benefits in diseases related to them.

## Methods

### Patients

Surgical specimens from 69 patients were analyzed. All surgical procedures were carried out at the Department of Urology, University of Tsukuba Hospital, Tsukuba, Japan. The tumor tissues were collected according to the institutional review board-approved protocols at the University of Tsukuba Hospital. Comprehensive informed consent was obtained from all participants. Clinical information was obtained from the medical records of patients. Normal testicular tissue specimens were obtained from the orchiectomy samples of prostate cancer patients before initiating hormonal therapy, and normal spermatogenesis was confirmed by hematoxylin and eosin staining of the tissue sections.

### Numbers of human testicular tissues used for immunohistochemistry

Five normal testes, and 64 testicular germ cell tumors consisting of 20 seminomas, 19 non-seminomas (8 embryonal carcinomas, 3 yolk sac tumors, 1 choriocarcinoma, and 7 teratomas) and 25 mixed tumors (10 with a seminoma component, and 15 without a seminoma component) were analyzed.

### Mice

Generation of the *CIRP*-KO mice was described previously^13^. *RBM3*-KO mice^59^ was kindly provided by Prof. T. Taniguchi, the University of Tokyo. The mutant mice were backcrossed to C57BL/6 J wild-type mice at least seven generations before being used in the experiments. *CIRP*-KO mice were mated to *RBM3*-KO mice to obtain *CIRP-RBM3* double KO mice. All mice were housed in standard cages at 20 °C under 12-h cycles of light and dark, and had ad libitum access to normal mouse chow and water. All animal experiments were reviewed and approved by the Animal Committee of Kyoto University. Experimental procedures involving animals and their care were conducted in conformity with institutional guidelines that complied with the Fundamental Guidelines for Proper Conduct of Animal Experiment and Related Activities in Academic Research Institutions under the jurisdiction of the Ministry of Education, Culture, Sports, Science and Technology, Japan.

### Cell lines and cell culture

Human U-2 OS, HEK293, and NC65 cell lines, and mouse NIH/3T3 and BALB/3T3-derived B22 cell lines, embryonic fibroblasts derived from wild-type and *CIRP*-KO mice, and lung fibroblasts from wild-type, and *CIRP-RBM3* double KO mice were grown in DMEM supplemented with 10% heat-inactivated bovine serum or fetal bovine serum (FBS). Human THP-1 cells were grown in RPMI-1640 medium supplemented with 10% FCS. Cell numbers were assessed by the colorimetric MTT assay (Promega) or by using a counting chamber under a microscope. Transfection of cells was performed with Lipofectamine-2000 or the calcium phosphate method.

For hypothermia experiments, a humidified CO_2_ incubator was used at 32 °C or 35 °C. In hypoxia experiments, O_2_ was tightly regulated at 1% employing a humidified three gas regulated CO_2_ incubator. UV irradiation was performed by using a Stratalinker 1800 UV crosslinker. Hyperosmotic stress involved addition of 400 mM sorbitol to PBS or addition of 60 or 160 mOsm NaCl to regular culture media. Hypotonic media contained 10 or 20% (volume/volume) of H_2_O. Generally, various stresses were given to cells in full confluency.

### Primers

The primer sets for quantitative PCR were as follows: for mouse actin, 5′-TCAGAAGGACTCCTATGTGG-3′ and 5′-TCTCTTTGATGTCACGCACG-3′; for mouse CIRP, 5′-AAGTGGTGGTGGTAAAGGACAG-3′ and 5′-ATGGAAGGACGATCTGGACG-3′; for mouse RBM3, 5′-AACACCGATGAACAGGCACTTGAAG-3′ and 5′-TAGCTCTCATCGCATCTGAGG-3′; for human SRSF12, 5′-TTGACTTCTACACTCGCCGC-3′ and 5′-ATTTGGCCTGGTGTTTTGCG-3′; for human SRSF11, 5′-CAGTCCAGATGTCGTCAGCA-3′ and 5′-GGGTTCTCGCTCCTGTTGATT-3′; for human SRSF10, 5′-CGACAATGATAGACCAAACTGC-3′ and 5′-CCTTTGGTCGCTTGAACTGC-3′; for human SRSF9, 5′-TACGTGGGGAACCTTCCGA-3′ and 5′-GCATCCTCTGCATCTCGGGG-3′; for human SRSF8, 5′-TCTGGGTCCTCCACTAGCTC-3′ and 5′-TCTTGGATCGCGACCTTGAC-3′; and for human SRSF7, 5′-CGAGGTATTTCCAATCCCCGT-3′ and 5′-TGGACTTTGATCGGCTGCTT-3′; for human SRSF6, 5′-AAAAAGTCGCTCCCGTTCCA-3′ and 5′-ACCGAGACCTGCTTCGAGAT-3′; for human SRSF5, 5′-GCGCAGTTGATTCGAGGAAG-3′ and 5′-TGGCCGCTGGATTTAGTCTC-3′; for human SRSF4, 5′-TGAAGAACGGATATGGTTTTGTGG-3′ and 5′-GTCCAGAACCGTAACTGCCA-3′; for human SRSF3, 5′-CCGGAGCAGGTCCCTTTCTA-3′; and 5′-ATCGGGACGGCTTGTGATTT-3′; for human SRSF2, 5′-CGGAGCCGCAGCCCTA-3′ and 5′-GGTCGACCGAGATCGAGAAC-3′; for human SRSF1, 5′-ATCTCATGAGGGAGAAACTGCC-3′ and 5′-GTAACTGCGACTCCTGCTGT-3′; for human TRPV4, 5′-GGCAACATGAGGGAGTTCATTAACTC-3′, and 5′-TTCTCCGTCAGGTAGTTGACAATGTG-3′; for human 18S rRNA, 5′-CTCAACACGGGAAACCTCAC-3′, and 5′-CGCTCCACCAACTAAGAACG-3′, and for human TRPA1, 5′-CCAAGCTGCATTTTCAGGTTCCAAAG-3′, and 5′-TGGCAGCAAAATGAATGGCTGTGCAC-3′.

### Plasmids

For constructing pFRT/sh-SRSF5 plasmids, the CMV promoter and poly(A) signal region of pcDNA5/FRT vector were replaced with tandem repeats of mutated H1 promoter^13^ and oligonucleotides expected to generate siRNA of the following sequences: 5′-GAACAGAAAATCGTCTTAT-3′. To down-regulate expression of TRPV4 and TRPA1, pPuro2/sh-TRPV4 and pPuro2/sh-TRPA1 plasmids, respectively, were constructed that contained the puromycin-resistance gene driven by the SV40 promoter and tandem repeats of mutated H1 promoter and oligonucleotides expected to generate siRNAs of the following sequence: sh-TRPV4, 5′-GATCCCCGAGGTGGAGGAAGAAGATCTTCAAGAGAGATCTTCTTCCTCCA CCTCTTTTTGGAAA-3′, and sh-TRPA1, 5′-GATCCCCGGAATCGCTTAAGGTGGTTTTCAAGAGAAACCACCTTAAGCGATTCCTTTTTGGAAA-3′. To overexpress human SRSF5 tagged with FLAG, pIRES/puro2/EF1alpha promoter vector was used.

### Reagents

The sources of reagents were as follows: A23187 (Tocris Bioscience, Bristol, UK), actinomycin D (Nacalai Tesque, Kyoto, Japan), BAPTA-AM (Dojindo Molecular Technologies, Inc., Kumamoto, Japan), cycloheximide (Nacalai Tesque), doxorubicin hydrochloride (Toronto Research Chem. Inc., North York, ON), Fura-2-AM (Molecular Probes, Carlsbad, CA), gadolinium chloride (Nacalai Tesque), GSK1016790A (Sigma-Aldrich), HC067047 (Tocris Bioscience), RN1734 (Tocris Bioscience), RN1747 (Tocris Bioscience), and ruthenium red (Tocris Bioscience). All other chemicals were purchased from Nacalai Tesque.

### Antibodies

Rabbit polyclonal antibodies recognizing the C terminus of mouse CIRP and mouse RBM3 were prepared as described^7, 8^. The sources of commercial antibodies were as follows: anti-ACTIN (mouse monoclonal, clone C4, Millipore), anti-BAX (rabbit polyclonal, #2772, Cell Signaling), anti-SRSF1/SF2/ASF (mouse monoclonal, sc-33652, Santa Cruz Biotechnology, Inc., Texas), anti-SRSF2/SC35 (goat polyclonal, sc-10252, Santa Cruz), anti-SRSF5/SRp40 (rabbit polyclonal, Sigma-Aldrich; rabbit polyclonal, MBL, Nagoya, Japan), anti-SRSF6 (rabbit polyclonal, Sigma-Aldrich), anti-p53 (rabbit polyclonal, sc-6243, Santa Cruz Biotechnology), anti-p73 (rabbit monoclonal, ab40658, Cambridge, UK), anti-mouse immunoglobulins/HRP (goat polyclonal, DakoCytomation, Glostrup, Denmark), anti-rabbit immunoglobulins/HRP (goat polyclonal, DakoCytomation), and FITC-conjugated goat anti-rabbit immunoglobulins (DakoCytomation).

### Western blot analysis

Five to ten million cells were washed with 1× PBS and lysed with lysis buffer (50 mM TrisHCl pH 7.5, 250 mM NaCl, 2 mM EDTA, 50 mM NaF, 0.1 mM Na_3_VO_4_, 0.5% Nonidet-P40, 1 mM DTT) and a cocktail of protease inhibitors for 120 min at 4 °C and centrifuged at 15,000 rpm for 30 min. The protein concentration of cell lysates was measured using the Bio-Rad DC Protein Assay kit (Bio-Rad Laboratories, Hercules, CA, USA) and 10–20 μg of proteins were resolved on 10–15% sodium dodecyl sulfate/polyacrylamide gel electrophoresis (SDS-PAGE), and electrotransferred to 0.45 μm PVDF membrane at 200 mA for 120 min at 4 °C. In many experiments membranes were horizontally cut between the 27 and 36 kDa markers, and the upper and lower portions were separately probed. Nonspecific binding sites were blocked by incubating the membrane for 1 h with 5% non-fat dry milk in 1× Tris-buffered-saline-Tween (TBST) (20 mM Tris-HCl pH 7.5, 500 mM NaCl, 0.1% Tween-20). Membranes were first incubated overnight with primary antibodies at 4 °C in 5% non-fat dry milk and then with 1 μg/ml horseradish peroxidase-conjugated secondary antibody in TBST with 5% non-fat dry milk for 1 h at 25 °C. After each incubation step, membranes were washed 3 times for 10 min with 1× TBST, and bands were revealed with a chemiluminescence reagent (Chemi-Lumi-One or Chemi-Lumi-One Super, Nacalai Tesque). Images were acquired with the ChemiDoc imaging system and quantification of protein bands was done with Image Lab v4.0 software (Bio-Rad Laboratories).

### Quantitative RT-PCR

Extraction of RNA was performed by using the Sepasol-RNA I Super G (Nacalai Tesque) as described by the manufacturer. Total RNA (1 μg) was reverse transcribed into first strand cDNA using the ReverTra Ace qPCR RT kit (TOYOBO Co., Osaka, Japan). The qPCR reaction was performed using THUNDERBIRD SYBR qPCR Mix (TOYOBO Co.) with the StepOne Plus Real-Time PCR System. Data were analyzed using the delta-delta Ct method. All experiments were performed in triplicate with a minimum of three independent experiments.

### Immunnohistochemistry

4-μm-thick paraffin sections of tissues fixed in buffered formalin were pretreated with 10 mM citrate buffer (pH 6.1) in a microwave oven for 5 min. Endogenous peroxidase activity was blocked with 0.3% H_2_O_2_ for 10 min. The sections were incubated with 10% FBS for 30 min to reduce nonspecific binding, followed by incubation with the primary antibody at 4 °C overnight. They were subsequently incubated with horseradish peroxidase–conjugated anti-rabbit immunoglobulin antibody for 30 min. The enzymatic reaction was developed in a freshly prepared solution of 3,3′-diaminobenzidine tetrahydrochloride using DAKO Liquid DAB Substrate-Chromogen Solution for 10 min at room temperature. The sections were then counterstained with hematoxylin. Mouse testes were fixed with paraformaldehyde, embedded in paraffin, and analyzed by immunostaining as above.

### Immunofluorescence microscopy

Cells cultured on glass coverslips were fixed with 4% paraformaldehyde in PBS, and permeabilized with 0.2% Triton X-100. After three washes with PBS containing 0.1% Tween20 (PBST), cells were soaked in blocking solution (PBST containing 4% bovine serum albumin) for 5 min and incubated with anti-SRSF5 or anti-CIRP antibody diluted with the blocking solution for 60–120 min. Cells were then washed three times with PBST and incubated with FITC-conjugated goat anti-rabbit immunoglobulin antibody for 60 min. Cells were washed three times, then mounted in 90% glycerol-PBS containing 0.1% *p*-phenylendiamine and 1% *n*-propylgalate. Specimens were observed using a fluorescence microscope.

### Real time TAQMAN RT-PCR assays of human *H-RAS* mRNAs

Real time TAQMAN RT-PCR assays were performed as described by García-Cruz *et al*.^60^. Total RNA was extracted from about 1 × 10^6^ cells using TRIZOL reagent (Life Technologies, Inc.). cDNA was reverse-transcribed from total RNA samples using SuperScriptIII® from Invitrogen. The resulting cDNA was amplified by PCR using Taq Man Assay primers with the Taq Man Universal Non-amperase PCR Master Mix and analyzed with a 7500 ABI PRISM Sequence Detector System according to the manufacturer’s instructions. mRNA expression was calculated from the relevant signals by normalization with respect to the signal for glyceraldehyde-3-phosphate dehydrogenase (GAPDH) mRNA expression. The assay numbers for exons E3-IDX p19 H-RAS (Hs00978053_g1), and E4A-E4B H-RAS total (Hs00978051_g1) and for GAPDH (HS99999905_m1 housekeeping) were supplied by Applied Biosystems Gene Expression Assays (Applied Biosystems). Assays were run with Taqman Universal.

### Ca^2+^ imaging

Ca^2+^ imaging was performed as described previously^51^. Briefly, cells were incubated with 2 μM Fura2-AM in a standard bath solution containing 140 mM NaCl, 5 mM KCl, 2 mM MgCl_2_, 2 mM CaCl_2_, 10 mM HEPES, and 10 mM glucose, pH7.4. The solution temperature in the chamber was monitored and adjusted with a temperature controller (TC-344B, Warner Instruments). The Fura2 ratiometric fluorescence (340:380 nm) measurements were recorded. The Ca^2+^ concentration (nM) was calculated as described previously^51^.

### Statistical analysis

Data are presented as the mean ± SEM. Statistical analyses were performed using unpaired or paired Student’s t test with or without the Welch correction. All statistical analyses were carried out using Prism v6.0 software or JMP10 software. A *P*-value of <0.05 was considered significant.

## Electronic supplementary material

Supplementary Information
