# Supplementary material for: TRPV4-dependent induction of a novel mammalian cold-inducible protein SRSF5 as well as CIRP and RBM3
Source: Sci Rep. 2017 May 23;7:2295. doi: 10.1038/s41598-017-02473-x (PMC5442135; doi:10.1038/s41598-017-02473-x)
Supplement: Supplementary file 1 — Supplementary Information [file 41598_2017_2473_MOESM1_ESM.pdf]

Supplementary Information

TRPV4-dependent induction of a novel mammalian cold-inducible protein SRSF5 as well as CIRP and RBM3

Takanori Fujita[1,2], Hiroaki Higashitsuji[1], Hisako Higashitsuji[1], Yu Liu[1], Katsuhiko Itoh[1], Toshiharu Sakurai[3], Takahiro Kojima[4], Shuya Kandori[4], Hiroyuki Nishiyama[4], Motoi Fukumoto[5], Manabu Fukumoto[5,6], Koji Shibasaki[7], and Jun Fujita[1,8,*]

[1]Department of Clinical Molecular Biology, Graduate School of Medicine, Kyoto University, Kyoto, Kyoto 606-8507, Japan. [2]School of Economics, Nagoya University, Nagoya, Nagoya 464-8601, Japan. [3]Department of Gastroenterology and Hepatology, Kindai University Faculty of Medicine, Osaka-Sayama, Osaka 589-8511, Japan. [4]Department of Urology, Faculty of Medicine, University of Tsukuba, Tsukuba, Ibaraki 305-8575, Japan. [5]Department of Pathology, Institute of Development, Aging and Cancer, Tohoku University, Sendai, Miyagi 980-8575, Japan. [6]Department of Molecular Pathology, Tokyo Medical University, Shinjuku-ku, Tokyo 160-8402, Japan. [7]Department of Molecular and Cellular Neurobiology, Gunma University Graduate School of Medicine, Maebashi, Gunma 371-8511, Japan. [8]Department of Rehabilitation Medicine, Biwako-Chuo Hospital, Otsu, Shiga 520-0834, Japan.

[*]Corresponding Author: Jun Fujita. 54 Shogoin Kawaharacho, Sakyo-ku, Kyoto 606-8507, Japan. Phone: +81-75-751-3753. E-mail: jfujita@virus.kyoto-u.ac.jp

Supplementary Figure S1. Induction of SRSF5 protein by various stresses. (a) Embryonic fibroblasts from C57BL/6J wild-type mouse were cultured at 37°C or 32°C for indicated times, and cell lysates were analyzed by western blot (upper panels). Band intensities relative to those at 37°C were determined after normalization to ACTIN levels (lower graphs, data indicate mean ± SEM; n = 4). **(b)** Human HEK293 and NC65 cell lines, mouse NIH/3T3 cell line and mouse embryonic fibroblasts (MEF) from *CIRP*- knockout (KO) mouse were cultured at 37°C or 32°C for indicated times, and analyzed by western blot. Relative band intensities after normalization to ACTIN expression are shown below each panel (representatives of 2 independent experiments each). **(c** and **d)** U-2 OS cells were cultured at 37°C for 8 h after exposure to indicated doses of UV (c) or in the presence or absence of 20 nM doxorubicin (Doxo) (d). Cell lysates were analyzed by western blot, and relative band intensities were determined after normalization to ACTIN (data indicate mean ± SEM; n = 4 for c and n = 3 for d). **(e)** NIH/3T3 and U-2 OS cells were cultured at 37°C under normoxia (20% O_2) or hypoxia (1% O_2) for 8 h, and analyzed by western blot. Relative band intensities were determined after normalization to ACTIN (data indicate mean ± SEM; n = 3 or 4 for NIH/3T3 and n = 4 for U-2 OS). **(f, g** and **h)** U-2 OS cells were cultured at 37°C or 32°C for 12 h in the presence of indicated doses of cycloheximide (CHX)(f), for 8 or 24 h with regular media (-) or media containing additional 60 (+) or 160 (++) mM NaCl (g), or for 8 h with regular media (Iso) or hypotonic media containing 10% (volume/volume) of H_2O (Hypo)(h), and analyzed by western blot. Relative band intensities were determined after normalization to ACTIN (data indicate mean ± SEM; n = 5 for f, n = 3 or 4 for g, and n = 3 for h). **(i)** U-2 OS cells were incubated with hypotonic media containing 10% (volume/volume) of H_2O (Hypo) at 37°C for indicated times, and then media were replaced with regular media (Iso). After total of 8 h incubation, protein levels were analyzed by western blot (left panels, representative results). Relative band intensities were determined after normalization to ACTIN (right graphs, data indicate mean ± SEM; n = 4). Statistical significance was determined by Student's *t*-test. *, P <0.05. **, P <0.01. ns, P >0.05.

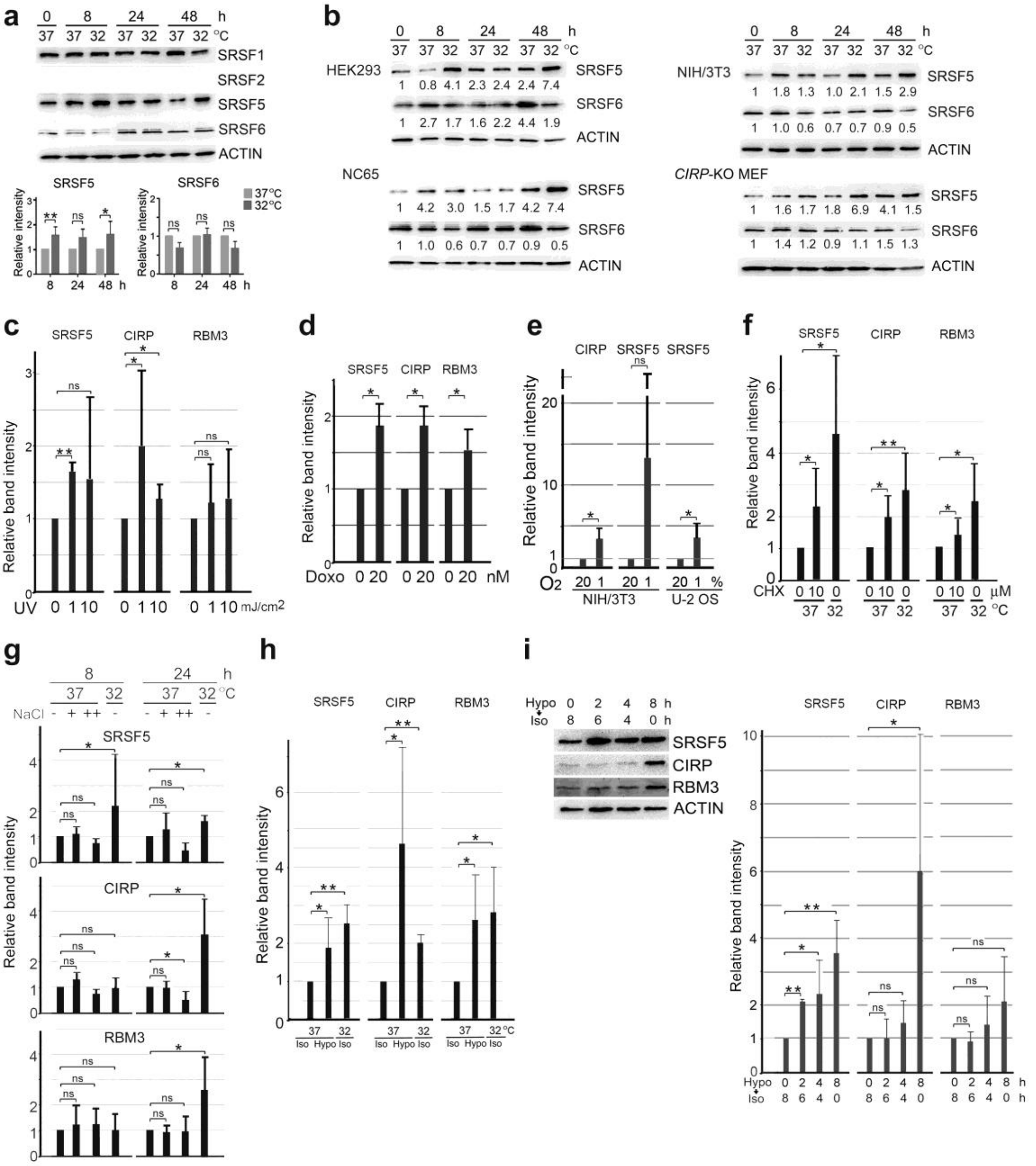
a
0 8 24 48 h
37 37 32 37 32 37 32 °C
SRSF1
SRSF2
SRSF5
SRSF6
ACTIN
SRSF5
SRSF6
37°C
32°C
Relative intensity
8 24 48 h
b
HEK293
1 0.8 4.1 2.3 2.4 2.4 7.4
1 2.7 1.7 1.6 2.2 4.4 1.9
NC65
1 4.2 3.0 1.5 1.7 4.2 7.4
1 1.0 0.6 0.7 0.7 0.9 0.5
NIH/3T3
1 1.8 1.3 1.0 2.1 1.5 2.9
1 1.0 0.6 0.7 0.7 0.9 0.5
CIRP-KO MEF
1 1.6 1.7 1.8 6.9 4.1 1.5
1 1.4 1.2 0.9 1.1 1.5 1.3
c
SRSF5 CIRP RBM3
Relative band intensity
UV 0 1 10 0 1 10 0 1 10 mJ/cm2
d
SRSF5 CIRP RBM3
Relative band intensity
Doxo 0 20 0 20 0 20 nM
e
CIRP SRSF5 SRSF5
Relative band intensity
O2 20 1 20 1 20 1 %
NIH/3T3 U-2 OS
f
SRSF5 CIRP RBM3
Relative band intensity
CHX 0 10 0 0 10 0 0 10 0 μM
37 32 37 32 37 32 °C
g
8 24 h
37 32 37 32 °C
NaCl - + ++ - - + ++ -
SRSF5
CIRP
RBM3
Relative band intensity
h
SRSF5 CIRP RBM3
Relative band intensity
37 32 37 32 37 32°C
Iso Hypo Iso Iso Hypo Iso Iso Hypo Iso
i
Hypo 0 2 4 8 h
Iso 8 6 4 0 h
SRSF5
CIRP
RBM3
ACTIN
Relative band intensity

Supplementary Figure S2. Subcellular localization and testicular expression of SRSF5 protein. **(a)** Western blot analysis of SRSF5 and CIRP in cell lysates and conditioned media from THP-1 cells cultured under hypothermia or hypoxia (1% O_2) for 24 h as indicated. Images after short and long exposures are shown. **(b)** Immunohistochemical (IHC) scores 0 to 3 for SRSF5 protein expression in human testicular tissue sections. **(c)** Hematoxylin and eosin (H&E) staining and IHC staining for SRSF5 of human testicular germ cell tumors. Representative results are shown. Scale bars, 50 μm. **(d)** IHC comparison of SRSF5 protein levels between seminomas with (M1, n = 10) and without (M0, n = 10) metastasis and embryonal carcinomas with (M1, n = 14) and without (M0, n = 12) metastasis (data indicate mean ± SEM). Statistical significance was determined by Student's *t*-test. ns, P >0.05.

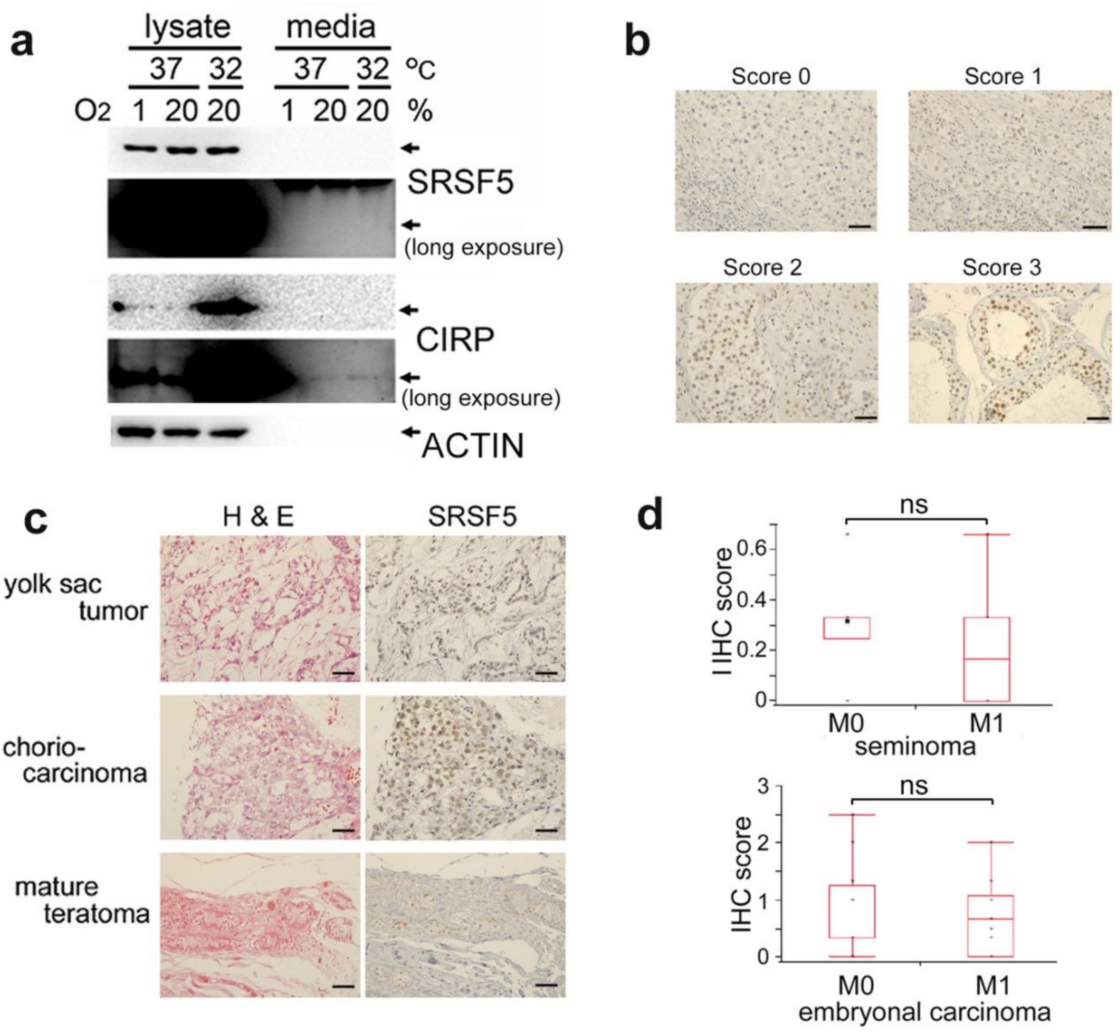

Supplementary Figure S3. Effects of SRSF5 on cell proliferation. (a) Stable transfectants of U-2 OS cells overexpressing SRSF5 (clones 1 and 2), vector alone, shRNA against SRSF5 (clones 1 and 2) or shRNA control were cultured at 37°C and analyzed by western blot for expression of SRSF5 protein. Relative band intensities after normalization to ACTIN expression are shown below the panel (representative of 2 independent experiments). **(b)** U-2 OS cells were cultured at 37°C in the presence (+) or absence (-) of 100 nM doxorubicin (Doxo) for 8 or 24 h, and analyzed by western blot. Relative band intensities were determined after normalization to ACTIN (data indicate mean ± SEM; n = 4). **(c)** Stable transfectants of U-2 OS cells overexpressing SRSF5 or vector alone were cultured at 37°C in the presence (+) or absence (-) of 100 nM Doxo for 10 h, and analyzed by western blot. Relative band intensities after normalization to ACTIN expression are shown below each panel (representative of 2 independent experiments). Statistical significance was determined by Student's *t*-test. *, *P* <0.05. **, *P* <0.01. ns, *P* >0.05.

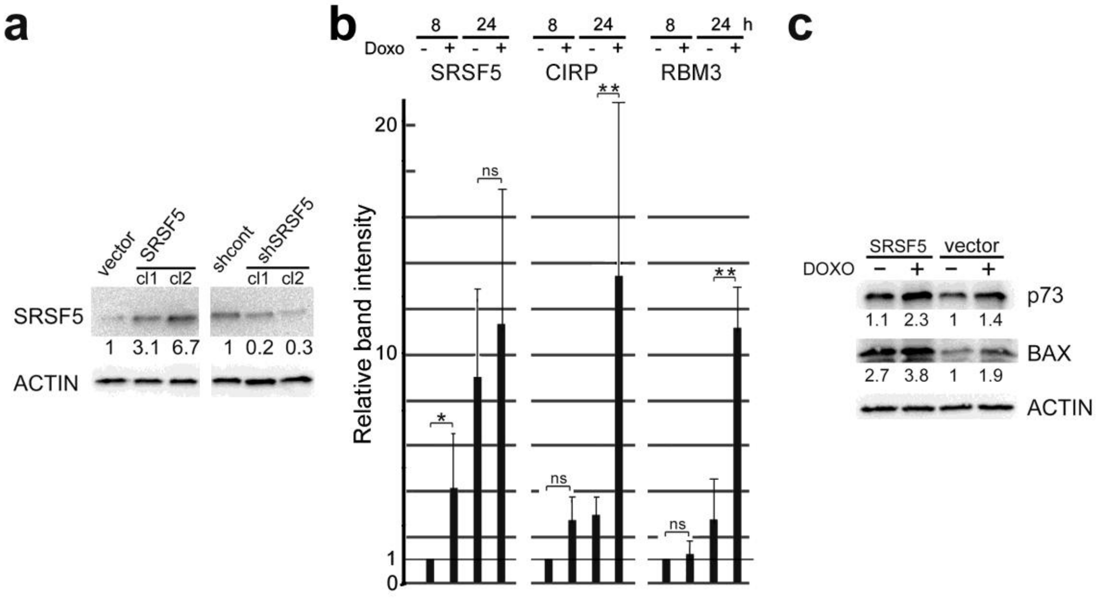

Supplementary Figure S4. Regulatory mechanisms of SRSF5 induction. (a) Growth curves of BALB/3T3 cells and B22 cells maintained at 37°C and transferred to 37°C or 32°C for indicated times (data indicate mean ± SEM; n = 3). **(b)** B22 cells were cultured at 37°C or 32°C for indicated times, and cell lysates were analyzed by western blot. Band intensities were determined after normalization to ACTIN and expressed as relative to those at 37°C (data indicate mean ± SEM; n = 3). **(c** and **d)** U-2 OS cells were incubated at 37°C or 32°C with isotonic regular media (0%) or hypotonic media containing 10% (volume/volume) of H_2O in the presence of 0 or 30 μM RN1734 for 8 h (c) or with indicated concentrations of RN1734 for 24 h (d), and analyzed by western blot. Relative band intensities were determined after normalization to ACTIN (data indicate mean ± SEM; n = 3). **(e)** U-2 OS cells were cultured at 37°C or 32°C in the presence of indicated concentrations of RN1734 for 8 or 24 h, and analyzed by western blot. Representative results (left panels). Relative band intensities were determined after normalization to ACTIN (right graphs, data indicate mean ± SEM; n = 3). **(f)** U-2 OS cells were cultured at 37°C or 32°C in the absence (-) or presence (+) of 30 μM RN1734 together with 20 nM doxorubicin (Doxo) for 8 h, and analyzed by western blot. Band intensities were determined after normalization to ACTIN and expressed as relative to those with Doxo at 37°C in the absence of RN1734 (data indicate mean ± SEM; n = 4). **(g, h** and **i)** U-2 OS cells were cultured in the absence (-) or presence (+) of 30 μM RN1734 at 37°C or 32°C for 8 h after exposure to indicated doses of UV (g), for 8 h under normoxia (-) or hypoxia (1% O_2, +) (h), or for 12 h in the presence of indicated doses of cycloheximide (CHX) (i), and analyzed by western blot. Representative results are shown in upper or left panels. Relative band intensities were determined after normalization to ACTIN (lower graphs, data indicate mean ± SEM; n = 3 for g and i, and n = 4 for h). Statistical significance was determined by Student's *t*-test. *, P <0.05. **, P <0.01. ns, P >0.05.

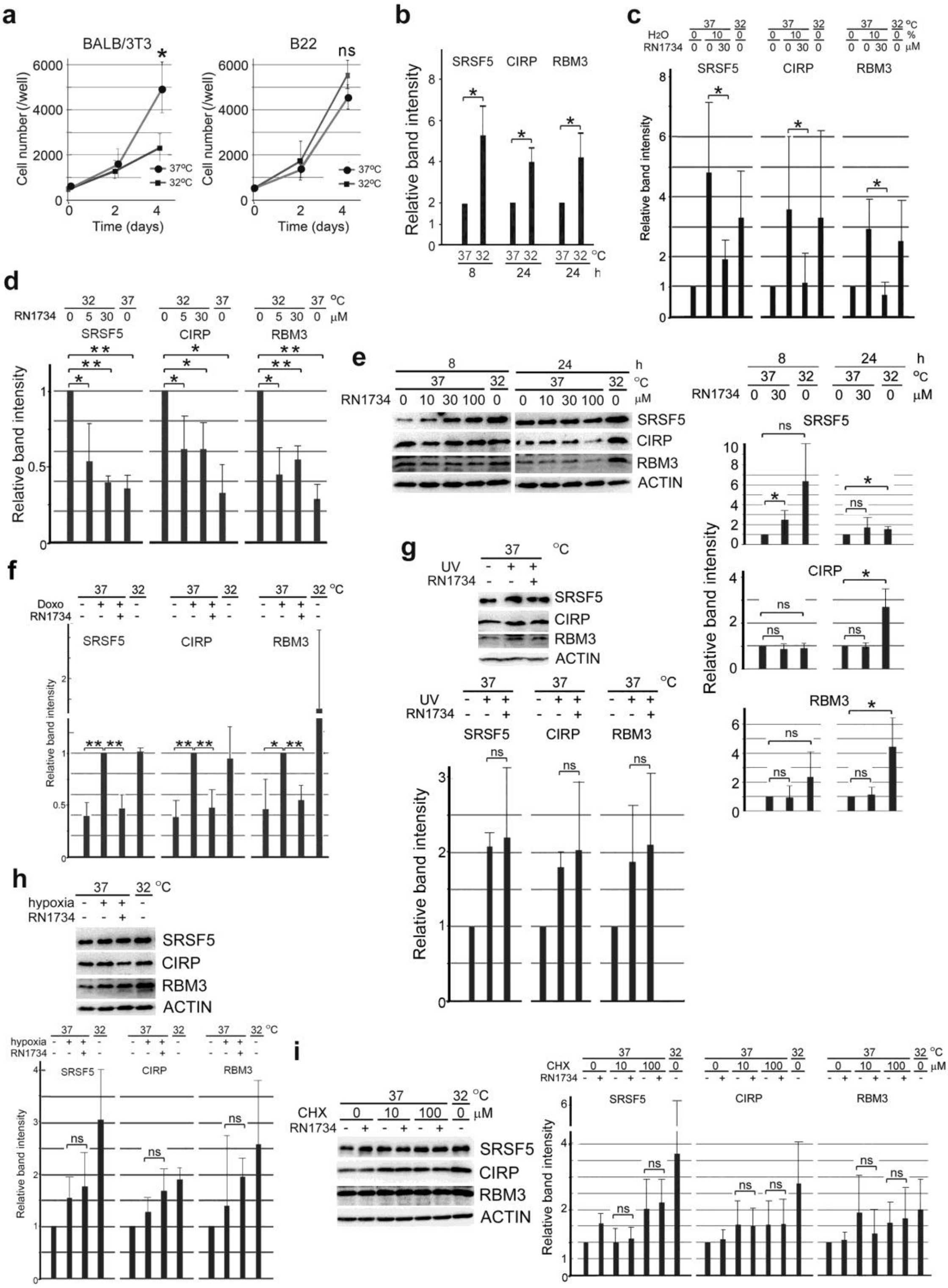
a
BALB/3T3
B22
Cell number (/well)
Time (days)
37°C
32°C
ns
b
SRSF5
CIRP
RBM3
Relative band intensity
c
H2O
RN1734
d
e
SRSF5
CIRP
RBM3
ACTIN
f
Doxo
g
UV
h
hypoxia
i
CHX

Supplementary Figure S5. TRPV4 ion channel activity and induction of CIPs.

(a and **b)** U-2 OS cells were cultured at 37°C or 32°C for 24 h in the presence of indicated doses of gadolinium chloride (Gd^{3+}) (a) or ruthenium red (RR) (b), and cell lysates were analyzed by western blot. Relative band intensities were determined after normalization to ACTIN (data indicate mean ± SEM; n = 3). **(c)** U-2 OS cells transiently transfected with plasmids expressing shRNA against TRPV4 (shTRPV4) or TRPA1 (shTRPA1) or vector alone (control) were cultured at 37°C for 16 h, and TRP mRNA levels relative to control were determined by quantitative RT-PCR after normalization to 18S rRNA (data indicate mean ± SEM, n = 3). **(d)** U-2 OS transfectants transiently expressing vector alone or shTRPV4 were cultured at 37°C or 32°C for 16 h, and analyzed by western blot. Band intensities were determined after normalization to ACTIN and expressed as relative to those at 37°C (data indicate mean ± SEM; n = 5). **(e)** U-2 OS cells transiently expressing vector alone (control) or shRNA against TRPA1 (shTRPA1) were cultured at 37°C or 32°C for 16 h, and analyzed by western blot. Relative band intensities after normalization to ACTIN expression are shown below each panel (representative of 2 independent experiments). **(f)** HEK293 transfectants transiently expressing vector alone or shTRPV4 were cultured at 37°C or 32°C for 16 h, and analyzed by western blot. Relative band intensities after normalization to ACTIN are shown below each panel (representative of 2 independent experiments). **(g)** U-2 OS cells were cultured at 37°C or 32°C for 6 h, and mRNA abundance at 32°C relative to that at 37°C was determined for TRPV4 by quantitative RT-PCR after normalization to 18S rRNA (data indicate mean ± SEM; n = 3). **(h** and **i)** U-2 OS cells were cultured at 37°C or 32°C for 24 h in the presence of indicated doses of RN1747 (h) or GSK1016790A (i), and analyzed by western blot. Relative band intensities were determined after normalization to ACTIN (data indicate mean ± SEM; n = 3). **(j** and **k)** U-2 OS cells (j) or HEK293 cells (k) were cultured at 37°C or 32°C for 8 h (j) or 24 h (k) in the presence of indicated doses of GSK1016790A, and analyzed by western blot. Relative band intensities after normalization to ACTIN are shown below each panel (representatives of 2 independent experiments each). **(l** and **m)** U-2 OS cells were cultured at 37°C or 32°C for 24 h in the presence of indicated doses of A23187 (l) or BAPTA-AM (m), and analyzed by western blot. Band intensities were determined after normalization to ACTIN and expressed as relative to those without chemicals at 37°C (left halves of l, and m) or 32 °C (right halves of l) (data indicate mean ± SEM; n = 3). **(n)** Quantification of intracellular Ca^{2+} concentrations by Fura-2 in HEK293 cells stably expressing rat TRPV4. Cells were cultured at 37°C or 32 °C for 24 h in the presence or absence of TRPV4 specific inhibitor HC067047 (data indicate mean ± SEM; n = 6). **(o)** U-2 OS cells were cultured at 37°C or 35°C for 8 or 24 h, and analyzed by western blot. Band intensities were determined after normalization to ACTIN and expressed as relative to those at 37°C (data indicate mean ± SEM; n = 4). Statistical significance was determined by Student's *t*-test. *, $P < 0.05$. **, $P < 0.01$. ns, $P > 0.05$.

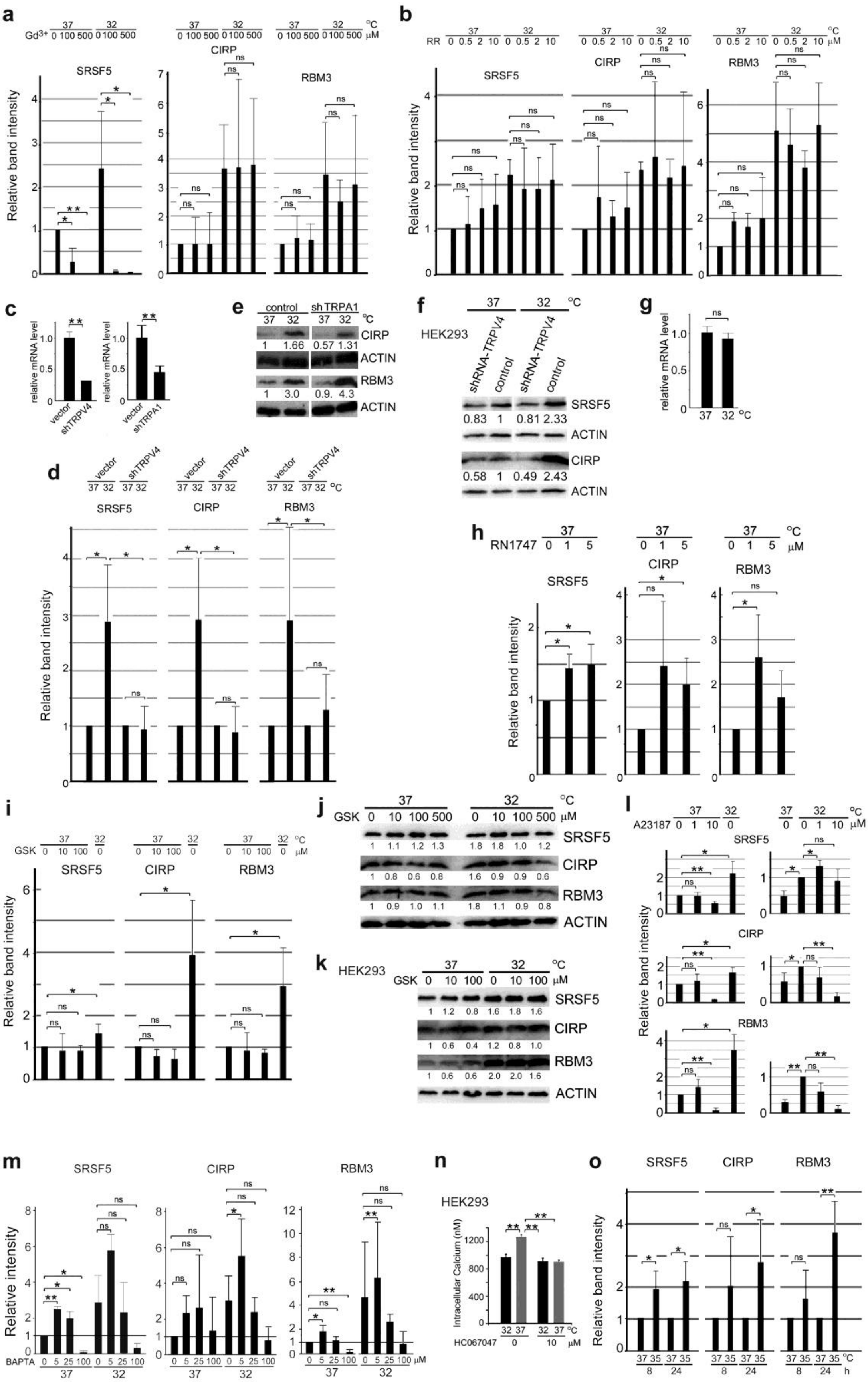
a
Gd3+
37 32
0 100 500 0 100 500
°C
μM
SRSF5
CIRP
RBM3
Relative band intensity
ns
b
RR
0 0.5 2 10 0 0.5 2 10
c
relative mRNA level
vector
shTRPV4
shTRPA1
e
control
shTRPA1
CIRP
1 1.66 0.57 1.31
ACTIN
RBM3
1 3.0 0.9. 4.3
f
HEK293
shRNA-TRPV4
control
SRSF5
0.83 1 0.81 2.33
CIRP
0.58 1 0.49 2.43
g
37 32 °C
d
h
RN1747
0 1 5
i
GSK
0 10 100 0
j
GSK 0 10 100 500 0 10 100 500 μM
1 1.1 1.2 1.3 1.8 1.8 1.0 1.2
1 0.8 0.6 0.8 1.6 0.9 0.9 0.6
1 0.9 1.0 1.1 1.8 1.1 0.9 0.8
k
HEK293
GSK 0 10 100 0 10 100 μM
1 1.2 0.8 1.6 1.8 1.6
1 0.6 0.4 1.2 0.8 1.0
1 0.6 0.6 2.0 2.0 1.6
l
A23187 0 1 10 0
m
Relative intensity
BAPTA 0 5 25 100 0 5 25 100
n
HEK293
Intracellular Calcium (nM)
HC067047 0 10 μM
o
37 35 37 35
8 24 h

Supplementary Figure S6. Full-length western blots.

(a to **g)** Full-length blots corresponding to Fig. 1. Indicated part (red box) or bands (arrows) are shown in Fig. 1b (a), Fig. 1d (b), Fig. 1e (c), Fig. 1f (d), Fig. 1g (e), Fig. 1h (f), and Fig. 1i (g). **(h** and **i)** Full-length blots corresponding to Fig. 3. Indicated parts (red boxes) or bands (arrows) are shown in Fig. 3c (h), and Fig. 3e (i). **(j** to **o)** Full-length blots corresponding to Fig. 4. Indicated bands (arrows) are shown in Fig. 4a (j), Fig. 4b (k), Fig. 4c (l), Fig. 4d (m), Fig. 4e (n), and Fig. 4f (o). **(p** to **w)** Full-length blots corresponding to Fig. 5. Indicated parts (white boxes) or bands (arrows) are shown in Fig. 5a (p), Fig. 5b (q), Fig. 5c (r), Fig. 5d (s), Fig. 5e (t), Fig. 5 f (u), Fig. 5g (v), and Fig. 5i (w). In most cases, membranes were cut and trimmed before incubation with antibodies.

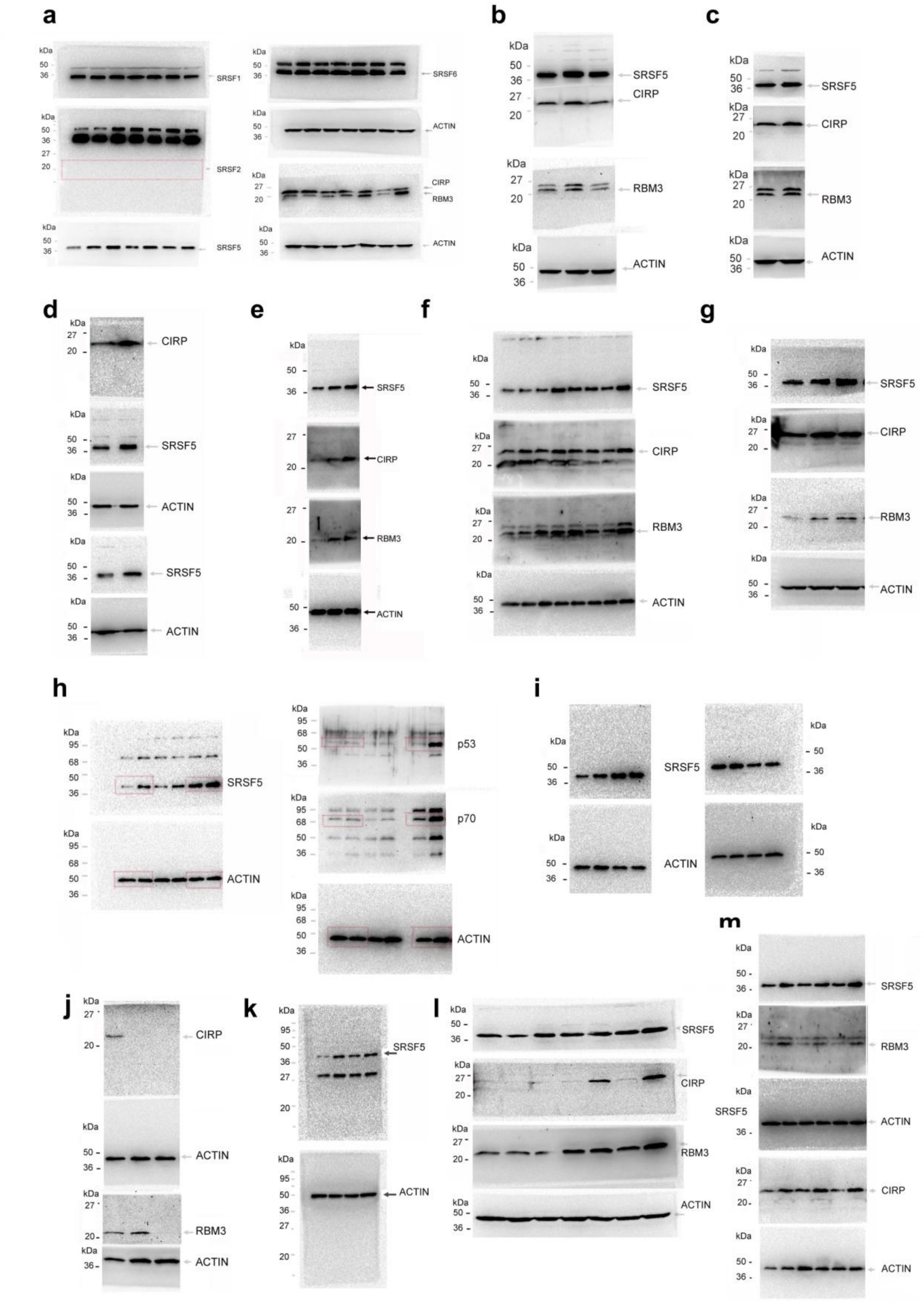
a
SRSF1
SRSF2
SRSF5
SRSF6
ACTIN
CIRP
RBM3
b
SRSF5
CIRP
RBM3
ACTIN
c
SRSF5
CIRP
RBM3
ACTIN
d
CIRP
SRSF5
ACTIN
e
SRSF5
CIRP
RBM3
ACTIN
f
SRSF5
CIRP
RBM3
ACTIN
g
SRSF5
CIRP
RBM3
ACTIN
h
SRSF5
ACTIN
p53
p70
i
SRSF5
ACTIN
j
CIRP
ACTIN
RBM3
k
SRSF5
ACTIN
l
SRSF5
CIRP
RBM3
ACTIN
m
SRSF5
RBM3
ACTIN
CIRP
kDa

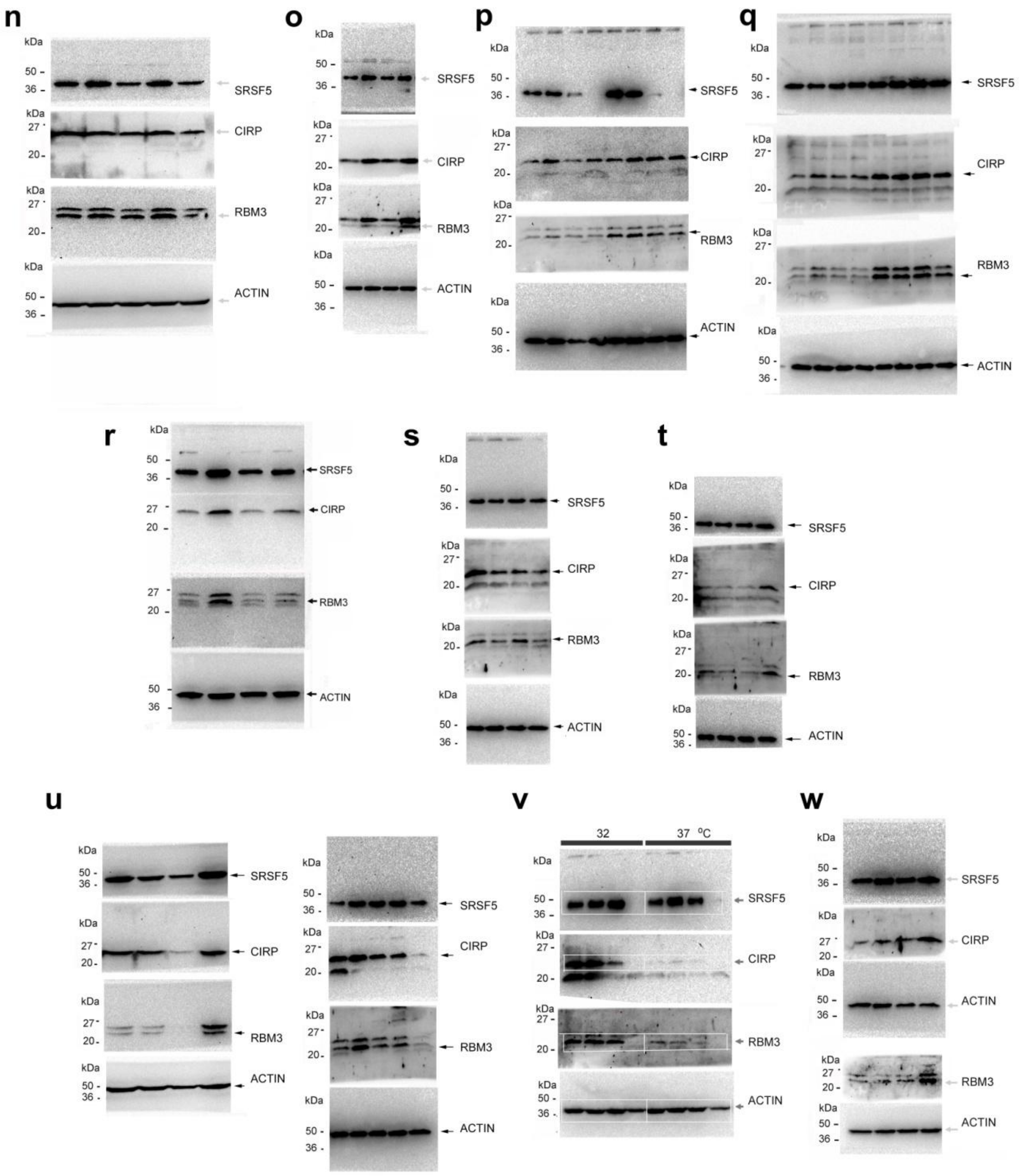
n
kDa
50
36
SRSF5
27
20
CIRP
RBM3
ACTIN
o
p
q
r
s
t
u
v
32
37 °C
w

Supplementary procedures

For analysis of cell culture supernatant, samples of 1.0 ml were centrifuged at 14,000 x g and concentrated to a volume of 10 to 60 µl using Amicon Ultra concentrators having a nominal cut-off of Mr = 50K (Millipore, Cork, IRL). The protein concentration of cell lysates was measured using the Bio-Rad DC Protein Assay kit (Bio-Rad Laboratories, Hercules, CA, USA) and subjected to western blot analysis.
